# Polyphenolic Compounds in the Stems of Raspberry (*Rubus idaeus*) Growing Wild and Cultivated

**DOI:** 10.3390/molecules29215016

**Published:** 2024-10-23

**Authors:** Ain Raal, Anni Vahtra, Oleh Koshovyi, Tetiana Ilina, Alla Kovalyova, Tõnu Püssa

**Affiliations:** 1 Institute of Pharmacy, Faculty of Medicine, University of Tartu, Nooruse Str. 1, 50411 Tartu, Estonia; annimaisla@gmail.com (A.V.); oleh.koshovyi@ut.ee (O.K.); 2 Department of Pharmacognosy and Nutriciology, National University of Pharmacy, Hryhoriy Skovoroda Str. 53, 61002 Kharkiv, Ukraine; allapharm@yahoo.com; 3 Department of Pharmaceutical Management, Drug Technology and Pharmacognosy, Ivano-Frankivsk National Medical University, Halytska Str. 2, 76018 Ivano-Frankivsk, Ukraine; ilyinatany86@gmail.com; 4 Food Hygiene and Safety Division, Chair of Veterinary Biomedicine and Food Hygiene, Institute of Veterinary Medicine and Animal Sciences, Estonian University of Life Sciences, Kreutzwaldi Str. 56/3, 51014 Tartu, Estonia; tonu.pyssa@emu.ee

**Keywords:** *Rubus idaeus*, stems, polyphenolic compounds, HPLC/MS analysis

## Abstract

The stems of *Rubus idaeus* L., a byproduct of the fruit–food industry, are known sources of bioactive compounds. The main objective of this study was to investigate the composition of polyphenolic compounds in *R. idaeus* stems. Seven cultivated raspberry varieties, thirteen garden samples, including five well-known raspberry varieties, and thirteen wild raspberry samples from different locations in Estonia were analyzed. The HPLC-MS method detected 62 substances, of which 42 were identified, 12 were tentatively identified, and 8 compounds remained unknown. Protocatechuic acid pentoside was dominant in most varieties and in all garden and wild raspberry samples. Dihydroxybenzoic acid hexoside 1, *p*-coumaroyl quinic acid 1, quercetin 4’-glucuronide, and *p*-coumaric acid glycoside were found in significant quantities. Correlations among the contents of individual compounds were established. When studying the dynamics of polyphenolic compound accumulation in, for example, the GR1 sample over a year, it was found that, in raspberry stems, the largest amount of them accumulated in April and slightly less in January and October. Investigating the dependence of the accumulation of polyphenols on the parts of the stem, it was found that the upper parts have the highest phenolic contents. Therefore, it is recommended to harvest approximately the upper third of the stem.

## 1. Introduction

*Rubus idaeus* L., or raspberries, or red raspberries, of the *Rosaceae* family, is a well-known plant with natural habitats in Europe, Asia, and North America, and it has been introduced in other regions of the world. It is one of the most famous cultivated berry crops [1,2]. In 2022, the world production of raspberries was 1.43 billion kg. The main producers were Mexico (18.33% of the world total), Serbia (12.25%), Poland (11.07%), the United States (8.07%), and Ukraine (3.54%) [3].

Its fruit provides the vitamins; minerals; fatty acids [4,5]; proteins; polyphenolic compounds [6,7,8], especially ellagitannins [9] and anthocyanins [10]; carbohydrates; and dietary fiber [11] needed for healthy nutrition in humans and animals [12,13,14,15]. Adding raspberries to starch-based foods does not alter the glycemic response [16].

Antioxidant [17,18,19,20,21,22,23], anti-inflammatory [24,25], antihypertensive [26], vasorelaxation [27], neuroprotective [28], and antimicrobial [29] activities have been determined for raspberry fruit. Its potential in modulating the risk of metabolic diseases, especially cardiovascular disease, diabetes, obesity, and Alzheimer’s disease—all of which have critical metabolic, oxidative, and inflammatory connections, has been confirmed [20]. It has been found that raspberry polyphenols may be a dietary route to slow down or alleviate neurodegenerative dysfunctions [21]. Flavonoids of *R. idaeus* had good therapeutic effect in a perimenopausal mouse model after their administration at high, medium, and low doses over time [22].

The chemical composition of raspberry leaves has been extensively studied. Polyphenolic compounds have been discovered [30,31,32,33,34,35,36,37], mainly hydrolyzable tannins (2.6% to 6.9%) [38], including gallotannins, which are esters of gallic acid and D-glucose [38,39]. Dimeric and tetrameric ellagitannins have also been identified, as well as flavonoids, such as kaempferol, kaempferol hexosides, quercetin, and quercetin glycosides [38], and phenolic acids, such as chlorogenic, gallic, ferulic, and caffeic acids [40]. In addition, terpenes, such as oxygenated monoterpenes, 1,8-cineole (50.8%), α-terpineol (5.2%), terpinyl acetate (3.7%), camphor (2.9%), and others [40]; carotenoids [8,35]; vitamins C and E; and minerals, such as calcium, magnesium, and zinc, have been identified. A monograph on Raspberry Leaf (ref.:2950) has recently been included in the Ph. Eur. [41].

In the EC, dry extract of *R. idaeus* leaves (solvent water) is a herbal remedy for the symptomatic relief of minor spasms associated with menstrual periods, for the symptomatic treatment of mild inflammation of the mouth or throat, and for the symptomatic treatment of mild diarrhea [42,43].

Raspberry leaf extract can significantly modulate platelet reactivity in whole blood. It affects platelet aggregation, possibly through modulation of the redox state, which depends on the oxidative activity of neutrophils [44]. Fatty acids and terpenoids account for the antifungal effect of raspberry leaves and stems against *Candida albicans* [45]. Experimental studies show that red raspberry leaf extract has antioxidant, antibacterial, and anti-inflammatory effects [46,47].

Stems are studied less than fruits, probably because of the wide use of fruit in human nutrition. However, the antioxidant, antimicrobial, and neutrophil-modulating activities of extracts of the herb *R. idaeus* have been established [48,49]. Crude aqueous extracts from the aerial part of raspberries exhibit antiparasitic activity against *Toxoplasma gondii* [50]. An antioxidant activity of raspberry stem and bark extracts has been found [51]. Research has shown that ethanolic extracts from the fruits, roots, stems, seeds, leaves, unripe fruits, and inflorescences of ‘Polka’ raspberry are effective against *Staphylococcus aureus*, *Listeria monocytogenes*, *Salmonella typhimurium*, *Bacillus subtilis*, *Enterococcus faecalis*, and *Pseudomonas aeruginosa* [52]. Raspberry stem extract has also been found to inhibit the activity of α-amylase and α-glucosidase, as well as to exhibit anti-AGE activity [53].

Raspberry stems have traditionally been used in Estonia as tea to relieve symptoms of colds and to reduce fever. In addition, raspberry stems have played important roles in relieving various pains (including rheumatism, joint, head, and abdominal pain), cough, menstrual ailments, diarrhea, indigestion, intestinal inflammation, internal bleeding, and anemia [54,55]. Tea from stems and leaves taken from the plant helps with acute respiratory diseases. The throat should be rinsed with a decoction of its leaves and stems to treat angina and laryngitis [56]. Baths made from its stems and twigs have been used for rheumatic pains, skin inflammations, and eczema [57].

In Estonian folk traditions, it is recommended to use different forms of raspberry plants primarily to lower fever in the case of a cold, and this is precisely because of their sweating effect. Raspberry stems are the most commonly used, followed by fruits and jams made from them [58]. In addition to lowering fevers, older people consider raspberry stalk tea a good treatment for coughs (especially closed, unproductive coughs), sore throats, bronchitis, and runny nose. It has been said that when suffering from tuberculosis, one should drink tea made from coarse raspberry stems. Also, for diabetes, raspberry stem tea is recommended, which is supposed to be drunk in the amount of 1 liter per day. Raspberry stalk tea is also a good remedy for relieving abdominal pain. In addition to the above, raspberry stem tea is important for women with painful menstruation. It has been suggested that rather younger shoots be used [58]. Since raspberries promote diuresis, they are also considered useful for bladder problems. Raw raspberries are eaten, half a liter daily, for nervous diseases and fever [58].

It is known that the contents of polyphenolic compounds and their compositions differ in wild and garden raspberries, as well as their varieties, and, in addition, depend on the stage of development and environmental conditions [51,59,60,61,62,63,64,65].

In farms that cultivate raspberries, pruning and thinning raspberry bushes are regular agrotechnical means [2]. Removed stems and shoots are production waste and are not used further. But, considering the experience of their use in traditional medicine, they can be an additional source of valuable biologically active compounds (BACs).

The aim of the study was to analyze the qualitative and quantitative contents of polyphenolic compounds in the stems of (1) raspberry cultivars (RCs), garden raspberry (GR), and wild raspberry (WR); (2) in different parts of the raspberry stems (five parts, from top to bottom); and also (3) to establish the dynamics of the contents of polyphenolic compounds in stems over 12 months.

## 2. Results

**Figure 1:**
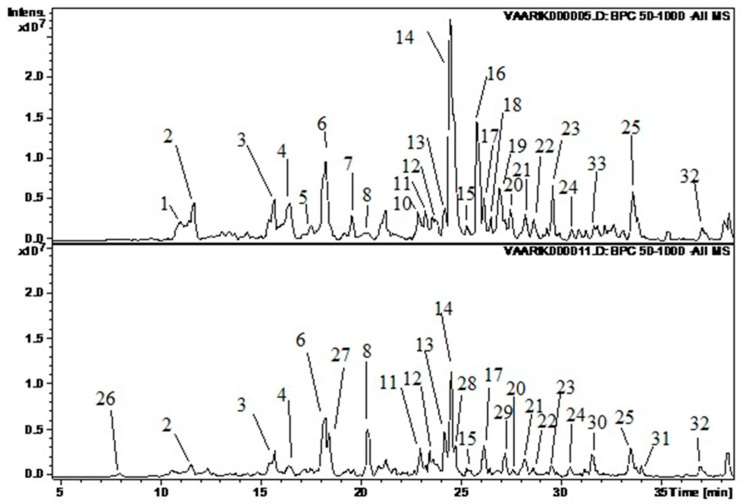
Illustration of the chromatograms (base peak chromatogram). The upper graph represents sample VA 4 and the lower WR 1. The following substances correspond to the peaks: 1—dihydroxybenzoic acid hexoside 2; 2—protocatechuic acid pentoside; 3—chlorogenic acid; 4—procyanidin B(2); 5—procyanidin B(3); 6—epicatechin; 7—*p*-coumaric acid glycoside; 8—*p*-coumaroyl quinic acid 2; 10—quercetin glucorhamnoside; 11—quercetin pentoside 1; 12—ellagic acid; 13—hyperoside; 14—quercetin 4’-glucuronide; 15—quercetin 7-glucuronide; 16—kaempferol glucoside; 17—isorhamnetin glucoside; 18—quercetin-3-(6”-(3-hydroxy-3-methylglutaryl)hexoside); 19—kaempferol glucuronide; 20—isorhamnetin/rhamnetin; 21—unknown 6; 22—acetylxyloside of ellagic acid; 23—isorhamnetin rhamnoside; 24—isorhamnetin rhamnoside 2; 25—unknown 10; 26—dihydroxybenzoic acid glucoside 1; 27—*p*-coumaroyl quinic acid 1; 28—isoquercetin; 29—isorhamnetin rhamnoside 1; 30—chlorogenic acid rhamnoside; 31—neochlorogenic acid rhamnoside; 32—isorhamnetin C-hexoside 2.

The results of the HPLC analysis of the raspberry stems are presented in Table 1 and Figure 1. For identification, the *m*/*z* of fragments of the MS/MS spectra of the substances were compared with data in the literature [66,67] and with data for standard substances.

**Figure 2:**
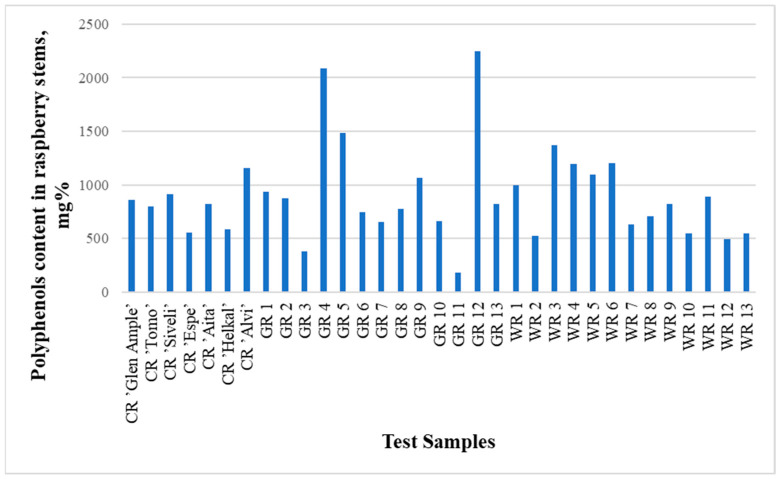
Graph comparing the total concentrations (areas of all peaks) of all substances studied in all 33 samples.

**Figure 3:**
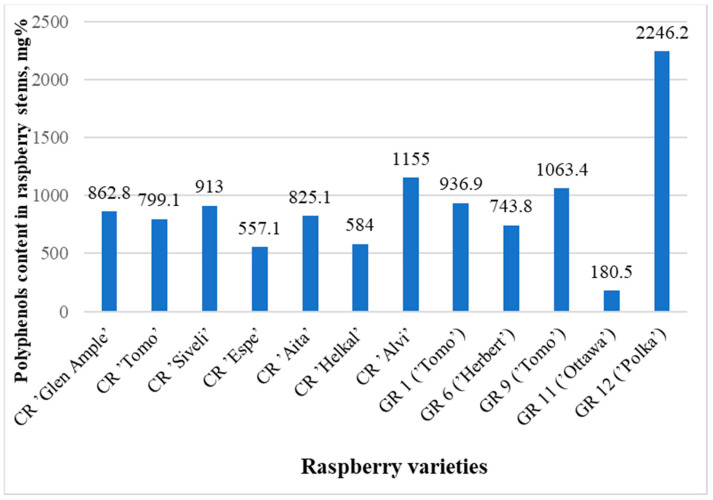
Graph comparing the total concentrations (areas of all peaks) of all substances in samples of known raspberry varieties.

The contents of identified phenolic compounds in the analyzed raspberry stems were from 180.5 mg% in sample GR11 to 2246.2 mg% in sample GR12 (Figure 2 and Figure 3, Table 2 and Table 3). The contents of polyphenols in the raspberry stems that grew in the wild (WR1-WR13) are presented in Table 4. Although the averages of the garden and wild raspberries’ results are significantly different (993.8 and 848.6, respectively), the sums of all quantified polyphenols are not statistically different (*p* = 0.77). The same can be said about the lack of difference between the results of these two groups of raspberries and the raspberry cultivars (*p* = 0.75 and *p* = 0.27, respectively) using the *t*-test.

In addition, a couple of samples were analyzed by positive ionization, which detected the presence of cyanidin hexoside, apparently either a glucoside or a galactoside. The mass of the corresponding positive molecular ion was 449, and the main fragment had a mass of 287.

**Figure 4:**
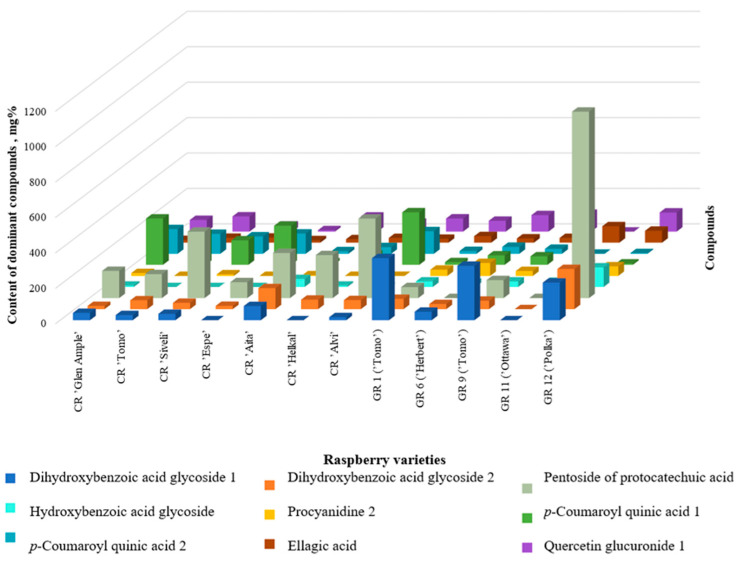
Comparison of the dominant polyphenolic compounds in raspberry cultivars.

It has been established that for most raspberry varieties, the dominant components are protocatechuic acid pentosidetechuic acid (five cultivars), *p*-coumaroyl quinic acid 1 (three cultivars), *p*-coumaroyl quinic acid 2, dihydroxybenzoic acid hexoside 1 and 2, and quercetin 4’-glucuronide (Figure 4).

In all garden and wild raspberry samples, protocatechuic acid pentoside protocatechuic acid was the absolute dominant. Dihydroxybenzoic acid hexoside 1, quercetin 4’-glucuronide, and *p*-coumaric acid glycoside were found in significant quantities.

**Figure 5:**
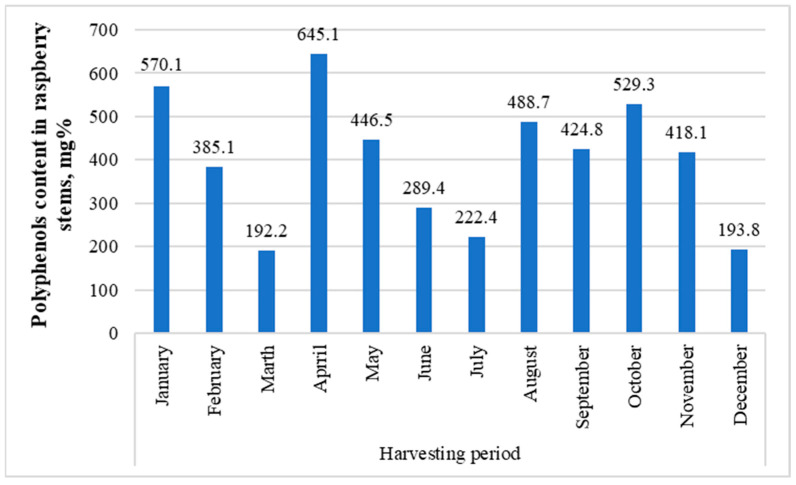
Total content dynamics of polyphenolic compounds in the GR1 sample over a year.

The raspberry bush used to study the contents’ dynamics over a year was also the following sample: GR 1. GR 1 was collected in mid-July 2016, and the July sample was collected in early July 2017. Interestingly, when comparing the two samples, the difference was significant (936.9 mg for GR 1 (Figure 3) and 222.4 mg for July (Figure 5)). This difference probably comes primarily from the fact that, for some reason, in all samples taken to study the year-round dynamics, dihydroxybenzoic acid hexosides 1 and 2, protocatechuic acid pentoside, dihydroferulic acid glycoside, and hydroxy-benzoic acid glycoside—which were present in the GR 1 sample and most others in fairly large quantities—are missing (Appendix A).

**Figure 6:**
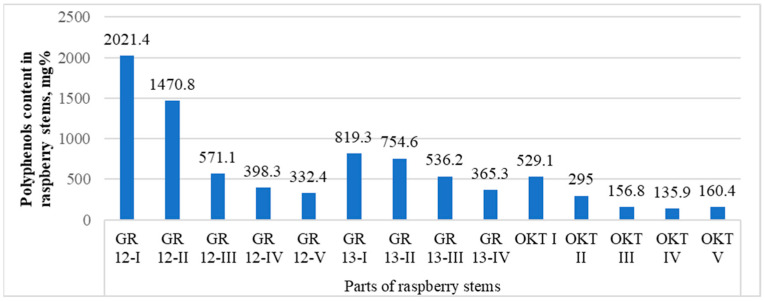
The contents of polyphenolic compounds in different parts of the raspberry stems, mg%. The parts of the stem, starting from the apex, are marked from numbers I to IV.

When studying the dependence of the accumulation of polyphenolic compounds in different parts of the stem on, for example, the GR 12, GR 13, and OCT samples (Appendix A, Table A1), it was found that the upper parts differed in their highest contents (Figure 6). When the contents of polyphenols were analyzed with the ANOVA test for the three comparison groups, it can be stated that, statistically, the results are the highest for the samples in #1, i.e., in the top part of the stems (*p* < 0.05). Therefore, harvesting from about the upper third of the stem is advisable.

When studying the correlations among the contents of individual compounds in the raspberry stems, a number of regularities were identified (Appendix B, Table A2 and Table A3).

## 3. Discussion

As a result of the HPLC analysis, 39 polyphenolic components were found in the raspberry stems. In addition, 12 substances were found, the identification of which gave grounds for caution and further investigation, and 11 unknown substances were fixed. In total, the peaks of 62 substances were detected. The total contents of polyphenolic compounds for individual cultivars such as ‘Glen Ample’ and ‘Polka’ differ from the data of other researchers [52,61].

The largest species compositions are distinguished by samples GR 1, GR 4, and GR 9, for which all 62 substances listed in the previous table were present. In addition to these, more than 56 substances (more than 90%) were found in samples WR 1, WR 3, WR 4, GR 2, GR 4, GR 5, GR 6, GR 7, GR 8, GR 12, CR ‘Aita’, CR ‘Glen Ample’, and CR ‘Siveli’ (Table 2, Table 3 and Table 4). Fewer than 43 substances (˂70%) were found in sample GR 11 (‘Ottawa’). Epicatechin, catechin, ellagic acid, ellagic acid 4-acetylarabinoside and acetylxyloside, quercetin, quercetin 3-(6”-(3-hydroxy-3-methylglutaryl)-hexoside 1, quercetin pentosides 1 and 2, rhamnetin/isorhamnetin, and isorhamnetin rhamnosides 1, 2, and 3 were detectable in all samples.

Dihydroxybenzoic acid hexosides 1 and 2, protocatechuic acid pentoside, chlorogenic acid, procyanidins 2 and 3, *p*-coumaroyl quinic acid 1 and 2, *p*-coumaric acid glycoside, dicaffeic acid derivative, hyperoside, quercetin rutinoside (rutin), quercetin 4’-glucuronide, isoquercetin, quercetin pentoside 3, quercetin hexoside malonate, kaempferol hexoside and glucuronide, isorhamnetin pentosides 1 and 2, isorhamnetin rhamnoside, isorhamnetin hexoside 1, and isorhamnetin rhamnosides 5 and 6, were detectable in over 80% of the samples. The detection of quercetin 3-glucuronide and quercetin glucosylrhamnoside (rutin) is consistent with previously published data on their presence in raspberry leaves [37]. The dominance of ellagic acid, the presence of protocatechuic and chlorogenic acids, hyperoside, quercetin-3-O-glucuronide, isoquercetin, monomeric catechin, and epicatechin, as well as dimeric proanthocyanidins—procyanidin B1 and B2, in raspberry shoots is confirmed by other scientists [51,52,60,61]. Hydroxybenzoic acid glucoside and neochlorogenic acid rhamnoside were present in less than 50% of the samples studied.

In general, the fluctuations between the months seemed to be considerably large, apparently due to weather conditions, in spring and autumn precisely (melting snow and freezing); the low concentrations in June and July during summer can be explained by the fact that the energy of the plant is primarily focused on the ripening of fruits.

It should be noted that, for some reason, all samples taken to study the year-round dynamics did not contain glycosides of dihydroxybenzoic acids 1 and 2, protocatechuic acid pentoside, dihydroferulic acid glycoside, and hydroxy-benzoic acid glycoside, which were present in the GR 1 sample and most others in sufficiently large quantities.

Procyanidin B(1) (2.6–13.5 mg%), procyanidin B(2) (47.0–271.0 mg%, highest in April, lowest in December), procyanidin B(3) (4.3–71.0 mg%, exceptionally high in August), catechin (1.9–23.9 mg%, highest in October), epicatechin (24.3–66.8 mg%, highest in May, April, and February), and *p*-coumaric acid glycoside (2.0–32.8 mg%, highest in January and April) were consistently found throughout the year, as well as quercetin pentoside 1 (0.7–2.0 mg%), ellagic acid (10.8–33.0 mg%), quercetin pentoside 2 (1.7–2.9 mg%), isoquercetin (1.3–49.0 mg%, highest in September, January, and October; lowest in December), isorhamnetin hexoside 1 (0.3–3.3 mg%), isorhamnetin pentoside 1 (0.3–1.4 mg%), isorhamnetin rhamnoside 1 (6.9–19.8 mg%), rhamnetin/isorhamnetin (0.2–1.4 mg%), ellagic acid acetylarabinoside (13.7–35.5 mg%, highest in April), ellagic acid acetylxyloside (9.4–32.2 mg%, highest in April), isorhamnetin rhamnoside 2 (0.7–2.3 mg%), and isorhamnetin rhamnoside 6 (1.1–3.9 mg%).

Of the other substances, chlorogenic acid and neochlorogenic acid, which were found in low concentrations, can be singled out only from August to November (chlorogenic acid also in January). Concentrations of *p*-coumaroyl quinic acids 1 and 2 were higher from August to November and in January, with the remaining months remaining several times lower. A similar phenomenon occurred with quercetin rutinoside (rutin) from August to October and in higher concentrations in January. The concentration of quercetin 4’-glucuronide was lowest in July, February, and March. Quercetin pentoside 3 was found in greater concentrations in January and September. An interesting sample was collected in July, which turned out to be the only one for which *p*-coumaroyl quinic acids 1 and 2, quercetin pentoside 3, quercetin hexoside malonate, chlorogenic acid rhamnoside, quercetin, and quercetin 4’-glucuronide were not detectable.

Also, with most individual substances, a smooth decrease in the concentration was noticeable, and in several cases it was deficient near the stem. For samples GR 13 and GR 12, the protocatechuic acid pentoside contents decreased from the top of the stem to the bottom. However, it was not detected at all in the OKT sample. Of the more significant changes, it should be pointed out that in sample GR 13, the largest amount of dihydroxybenzoic acid hexoside was found in the II quarter (almost three times more than the next), followed by III and I, and the lowest was still close to the stem. However, for the same substance in the GR 12 sample, the lowest level was found in the middle part (III), which then rose slightly as it moved to both sides. Hydroxybenzoic acid hexoside was uniformly found at around 20 mg% in the first three parts of GR 13, with 8.7 mg% in the stem part. For the same substance in the GR 12 sample, the highest level was found in the part II of the stem; lower levels in I and IV; and in III and V, it was undetected. In the second part of the stem, GR 12 also had higher levels of both catechin and epicatechin, but for GR 13 and OKT, they decreased evenly from the apex to the stem. A kind of dynamics appeared with procyanidins, which were the highest in part II of the GR 12 sample, and for the OKT sample they fell smoothly but then rose again in parts IV and V. The levels in the GR 13 sample were relatively constant in each section but still slowly decreased. The differences may have arisen for the parts of the stems due to their different lengths.

For the remaining substances, the changes were either barely noticeable or decreased according to the expected dynamics, being the highest at the peak and the lowest near the stem. Apparently, in the lower part of the stem, substances had lower concentrations, since on the stem side, it was woodier. Many substances, which were also not originally present in very high concentrations, were absent when close to the strain.

As a result of the data analysis (Table 2, Table 3 and Table 4, Appendix B: Table A2 and Table A3), quite strong correlations were found among the contents of the biologically active substances, and the Pearson coefficients confirm this. The correlation coefficients between the contents of procyanidins and catechins were r = 0.60–0.93; procyanidins and flavonoids, r = 0.60–0.73; derivatives of benzoic and ellagic acids, r = 0.60–0.70; individual hydroxycinnamic acids, r = 0.70–0.97; hydroxycinnamic acids and flavonoids, r = 0.60–1.00; benzoic acid derivatives and flavonoids, r = 0.62–0.84; ellagic acid derivatives and flavonoids, r = 0.61–0.88; and individual flavonoids, r = 0.60–0.97.

An absolute positive correlation was established between the contents of neochlorogenic acid rhamnoside–isorhamnetin rhamnoside 7 (r = 1.0). Very strong correlations (r = 0.97) were established for pairs of compounds such as quercetin 3-glucuronide-glucoside–isorhamnetin rhamnoside 7; *p*-coumaroyl quinic acid 1–*p*-coumaroyl quinic acid 2; and chlorogenic acid rhamnoside–neochlorogenic acid rhamnoside, as well as for dicaffeoyl quinic acid–isorhamnetin rhamnoside 7 (r = 0.94), procyanidin B(2)–epicatechin (r = 0.93), quercetin 3-glucuronide-glucoside–quercetin hexoside malonate, isoquercetin–isorhamnetin rhamnoside, quercetin–isorhamnetin rhamnoside 7 (r = 0.92), and isorhamnetin rhamnoside 1–isorhamnetin rhamnoside 6 (r = 0.91).

There were strong inverse correlations between pairs of compounds such as quercetin pentoside–isorhamnetin rhamnoside 7 (r = −1.0), isorhamnetin rhamnoside 1–neochlorogenic acid rhamnoside (r = −0.82), and neochlorogenic acid–quercetin pentoside (r = −0.80); and there were moderate inverse correlations between pairs of compounds such as neochlorogenic acid rhamnoside–isorhamnetin rhamnoside 6 (r = −0.78) and hydroxybenzoic acid hexoside–isorhamnetin rhamnoside 7 (r = −0.74) (Appendix B).

Phenolic compounds are known to have an adaptive function in plant life. Many works are devoted to studying the relationships between the accumulation of phenolic compounds and the duration of the light period, elemental composition of the soil, humidity, and altitude above sea level. We took the average data for the contents of the biologically active substances in 33 different cultivars of the species *R. idaeus*. Therefore, the revealed correlations among the different groups of biologically active substances characterize the genotypic correlations of the substances of the species.

The presence of positive, strong correlations indicates the conjugated biosynthesis and accumulation of these compounds in the 33 samples of stems of *R. idaeus* L. varieties, which confirm the genotypic relationships of these compounds, characteristic of this species.

## 4. Materials and Methods

### 4.1. Raw Materials

The work considers both garden varieties of raspberries and specific varieties of crops (Table 1). The varieties of the stems obtained from people’s home gardens are largely unknown. Brief descriptions of the varieties studied in this work (EMÜ, 2017, Neeva Garden, 2014) and their photos are provided in Appendix C.

The following raspberry stems used for the research were collected in the summer of 2016: 7 cultivated raspberry varieties (CR1-CR7) from the Polli garden of the EEC Horticultural Research Center; 13 from different home gardens (GR1-GR13), including five known raspberry varieties; and 13 samples from wild raspberries (WR1-WR13) in different regions of Estonia. Thus, a total of 33 samples of raspberry stems from different locations of growth were analyzed. Most of the samples were from Southern Estonia. Nineteen samples were collected from Viljandi County, four from Lääne County, four from Valga County, three from Tartu County, two from Ida-Viru County, and one from Rapla County (Table 5, Appendix C). The top parts, 20 cm long, were collected from stems for examination. To study the dynamics of the contents of polyphenolic compounds over a year, a single sample was collected every month from the same bush (sample GR1, apex parts, 20 cm long). Three raspberry bushes (GR12, GR13, and the October samples) were used as the samples to determine the contents of substances in the different parts of raspberry stems. From the bushes, stems as similar in length as possible were cut from the ground, and divided into five equal parts. The collected materials were stored in a refrigerator at −18 °C and analyzed immediately after defrosting. The losses upon drying the samples were measured according to the *European Pharmacopoeia*’s method (chapter 2.2.32) [68].

### 4.2. Preliminary Test to Determine a Suitable Solvent

**Figure 7:**
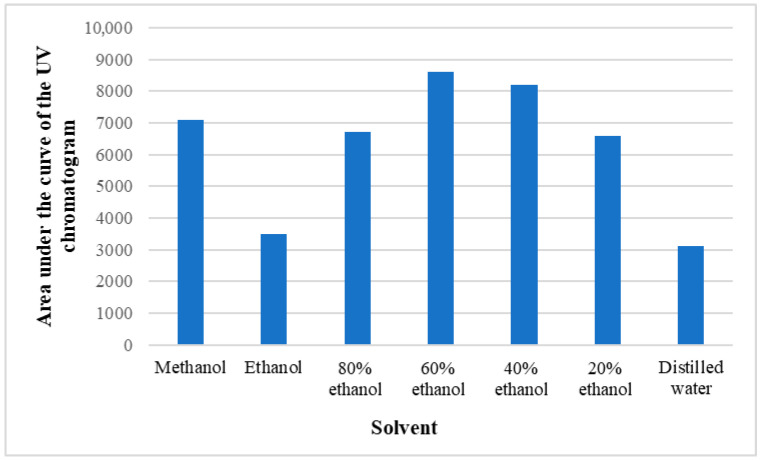
Areas of UV chromatograms obtained with different solvents.

Preliminary tests were conducted with different ethanol concentrations (20–80%), methanol, and distilled water to find the most suitable solvent for extraction of the phenolic compounds under investigation. In doing so, the base area of the HPLC UV chromatogram was estimated at 280 nm, where most of the phenolic substances absorb radiation, and it was concluded, based on both the qualitative and quantitative contents of the substances, that it is optimal to use 60% ethanol for the study of polyphenols (Figure 7).

### 4.3. Extraction and HPLC/MS Analysis of Polyphenolic Compounds in Raspberry Stems

To extract the polyphenols, raspberry stems were chopped into 1–2 mm long pieces with scissors, 0.50 g was weighed into a test tube, and 60% ethanol/water (*v*/*v*) was added to 10 mL. The samples were allowed to sit for 24 h with occasional slight shaking, and then the samples were filtered through a paper filter and centrifuged at 6000 rpm for 10 min.

An Agilent 1100 Series LC/MSD Trap-XCT with an ESI ionization unit was used. The blocks included an autosampler, solvent degasser, binary pump, column in the thermostat, and UV-Vis diode array detector. The column was a Zorbax 300SB-C18 (2.1 mm × 150 mm) with a particle diameter of 5 μm. HPLC 2D ChemStation software (01.11) was used in combination with the ChemStation Spectral SW module to control the process. A total of 5 μL of the test solution was injected into the column, the elution time was 50 min, the UV-Vis diode detector operated in the wavelength range of 190–530 nm, and the temperature of the column was kept at 35 °C. The analytes were separated using a C18 reversed-phase column and an ascending linear gradient of an aqueous 0.1% formic acid solution (eluent A) and acetonitrile (eluent B). Polyphenols were identified by an ion trap with an MS/MS detector using the negative ionization mode (Table 2, Figure 2). The particle mass-to-charge ratio range (*m*/*z*) under study was 50–1700, with a target *m*/*z* of 1000. The flow rate was 0.3 mL/min.

To determine the quantitative contents of polyphenols, solutions of a certain concentration of 96% ethanol were prepared from the standard substances and chromatographed under the same conditions as rhubarb stem extracts, with the difference that the target mass of the characteristic substances was 700 *m*/*z*. With the help of a computer program, the base areas of the characteristic peaks were determined, and a calibration graph was prepared for each standard substance. The following standards were used: quercetin glucoside (Sigma-Aldrich, St. Louis, MO, USA), ≥90%-HPLC-purity quercetin galactoside (Sigma-Aldrich), ≥97%-HPLC-purity myricetin (Sigma-Aldrich), ≥96%-HPLC-purity kaempferol (Sigma-Aldrich), ≥90%-HPLC-purity quercitrin (Alpha-Aesar, Haverhill, MA, USA), and caffeic acid (Sigma-Aldrich). A similar methodology was used in our previous studies [69].

By comparing the basal areas of the characteristic peaks of the standards with those of raspberry, the contents of substances in 1 g of herbal drug was calculated. Since some of the standard polyphenols were in the form of aglycones (for example, myricetin and kaempferol) but in the plant material present as glycosides, a coefficient was used for the aglycone, with the help of which the concentration of glycoside was obtained. The coefficient (x) was calculated according to the following formula:

$$x=\frac{glycoside\;molecular\;weight}{aglycone\;molecular\;weight}$$

The content of a particular substance in the dried herbal drug was calculated according to the straight formula for the calibration graph of the characteristic substance, as follows:

$$x=\frac{\left(y-b\right)}{m}\times 10\times 20$$

where

x—substance’s content in dried herbal drug (mg%);

y—area under the peak of the tested substance (area units);

b—calibration straight intersection with the *y*-axis;

m—straight ascent;

10—transition coefficient from peak areas to the concentration in µg/mL into mg%;

20—drug:solvent ratio (1:20)—transition coefficient of the concentration of the analyzed extract, in µg/mL, to dried herbal drug, in µg/g.

## 5. Conclusions

The compositions of the stems of wild and garden raspberries were compared for the first time in this work. The HPLC-MS method detected 62 substances, of which 42 compounds were identified, 12 were suspected, and 8 were unknown.

The largest amount of polyphenolic compounds was found in the garden raspberry sample GR12 (‘Polka’)—2246.2 mg%—and in the sample GR4—2089.6 mg%.

The main polyphenolic ingredients of raspberry stems are protocatechuic acid pentosidetechuic acid, *p*-coumaroyl quinic acid 1, *p*-coumaroyl quinic acid 2, dihydroxybenzoic acid hexosides 1 and 2, and quercetin 4’-glucuronide. There were no significant differences in the chemical compositions of the garden and wild raspberries.

The variety of raspberry and its place of growth significantly impact the composition of substances contained in stems. Over the year, the largest amounts of them accumulated in the raspberry stems in January (570.1 mg%), April (645.1 mg%), and October (529.3 mg%). Therefore, these months are the most optimal for procuring raw materials.

When studying the correlations among the contents of individual compounds in the raspberry stems, a number of regularities were established. An absolutely positive correlation was established between the contents of neochlorogenic acid rhamnoside and isorhamnetin rhamnoside 7 (r = 1.0) and an inverse correlation between quercetin pentoside and isorhamnetin rhamnoside 7 (r = −1.0).

Various phenolic substances are more numerous at the apex of the raspberry stem than near the stem, and the concentrations of these substances are also higher at the apex.

## Figures and Tables

**Table 1:** Phenolic compounds detected in raspberry stems by HPLC-MS in negative ionization.

Rt	*m*/*z* (M-H)^−^	*m*/*z* of Main Collision Fragments	Compound
8.1	315	153;109	Dihydroxybenzoic acid hexoside 1
11.8	315	297;153;109	Dihydroxybenzoic acid hexoside 2
12.0	285	153;109	Protocatechuic acid pentoside
13.8	299	179;137;135	Hydroxybenzoic acid hexoside
14.7	577	559;451;425;407;289	Procyanidin B(1)
14.8	357	195;339	Dihydroxyferulic acid glucoside
15.1	289	245;205;179;125	Catechin
16.0	353	191;179;135	Chlorogenic acid
16.7	577	559;451;425;407;289	Procyanidin B(2)
17.7	577	559;451;425;407;289	Procyanidin B(3)
18.2	353	191;179;135	Neochlorogenic acid
18.5	639	463;301	Quercetin 3-glucuronide-glucoside
18.6	289	245;205;179;125	Epicatechin
18.8	337	191;163;173;301	*p*-Coumaroyl quinic acid 1
19.6	325	163;119;289	*p*-Coumaric acid hexoside
20.7	337	191;163;173;301	*p*-Coumaroyl quinic acid 2
23.1	609	301;302;431;179	Quercetin glucorhamnoside
23.2	595	463;343;301;300;179	Quercetin pentohexoside (rumarin)
23.4	433	301;300;151	Quercetin pentoside 1
23.9	301	229;257;185;284	Ellagic acid
23.9	433	300;302;387;161	Quercetin pentoside 2 *
24.4	463	301;179;343;271	Quercetin galactoside (hyperoside)
24.6	477	301;179	Quercetin 4’-glucuronide
24.6	567	341;329;521;279	Unknown 1
24.6	609	301;343;271;179	Quercetin rutinoside (rutin)
24.8	499	475;463;489	Unknown 2
24.9	463	301;271;179;355;161	Quercetin glucoside (isoquercetin)
25.6	477	301;323;221;179;161	Quercetin 7-glucuronide
26.0	433	300;301;151;179	Quercetin pentoside 3
26.2	447	285;255	Kaempferol hexoside
26.2	505	463;301;300;271	Quercetin acetylhexoside 1
26.2	607	463;301;151;545;505	Quercetin 3-[6”-(3-hydroxy-3-methylglutaryl)-hexoside] 1
26.4	477	315;153;433	Isorhamnetin hexoside 1
26.8	447	315;300	Isorhamnetin pentoside 1
26.8	607	463;301;151;545;505	Quercetin 3-[6”-(3-hydroxy-3-methylglutaryl)-hexoside] 2
26.9	505	461;301;300;271;179	Quercetin acetylhexoside 2
27.0	461	285;323;357;175	Kaempferol glucuronide
27.2	475	301;300;315;153	Isorhamnetin rhamnoside 1
27.3	477	301	Quercetin 3-glucuronide
27.4	567	521;179;559;341;390	Dicaffeic acid derivative *
27.5	447	315;300	Isorhamnetin pentoside 2
27.7	315	300;301;271;153	Rhamnetin or isorhamnetin *
28.2	505	323;389;301;179;161	Acetyl hexoside
28.3	475	415;300;301;185	Ellagic acid acetylarabinoside *
28.3	571	523;345;357;195;493	Quercetin-3-glucuronide
28.7	475	300;301;323	Ellagic acid acetylxyloside *
28.7	515	353;191;179;317;299	Dicaffeoyl quinic acid
29.6	461	301;315;159;179;151	Isorhamnetin rhamnoside 1 *
29.8	571	523;345;357;195;493	Unknown 3
30.4	489	315;429;300	Isorhamnetin rhamnoside 2 *
31.6	499	353;173;203;255	Chlorogenic acid rhamnoside *
31.7	489	315;429;300	Isorhamnetin rhamnoside 3 *
31.8	301	151;179;257;211	Quercetin
32.1	517	300;457;179	Unknown 4
32.6	489	315;429;300	Isorhamnetin rhamnoside 4 *
33.4	585	537;359;330;223	Unknown 5
33.5	517	300;457;179	Unknown 6
33.8	585	537;359;330;223	Unknown 7
34.1	499	353;173;460;256	Unknown 8
35.8	531	471;300;314;411;456	Isorhamnetin C-hexoside 1 *
36.9	531	315;300;411;471	Isorhamnetin *C*-hexoside 2 *1
38.0	531	315;300;471;411	Isorhamnetin *C*-hexoside 3 *

* In the case of these substances, doubts arose because of the non-overlap of some fragments, the retention time, or the identity of the substance came mainly from the literature.

**Table 2:** Polyphenolics in the stems of raspberry cultivars, mg% (mg in 100 g).

Compound	CR ‘Glen Ample’	CR ‘Tomo’	CR ‘Siveli’	CR ‘Espe’	CR ‘Aita’	CR ‘Helkal’	CR ‘Alvi’
Dihydroxybenzoic acid hexoside 1	41.5	29.3	36.1	-	80.5	-	17.5
Dihydroxybenzoic acid hexoside 2	18.1	49.9	35.7	18.5	118.5	53.5	51.1
Protocatechuic acid pentoside	153.6	135.1	375.0	89.8	254.8	242.7	448.8
Hydroxybenzoic acid hexoside	8.2	-	-	-	45.3	8.3	-
Procyanidin B(1)	2.4	-	-	-	-	-	-
Dihydroxyferulic acid glycoside	-	-	2.0	-	-	-	-
Catechin	7.8	7.7	15.2	2.3	7.8	6.8	2.7
Chlorogenic acid	5.6	-	3.0	7.2	1.2	1.5	5.2
Procyanidin B(2)	16.3	-	8.9	-	3.6	-	-
Procyanidin B(3)	4.8	5.9	3.2	2.2	8.6	5.1	2.5
Neochlorogenic acid	1.0	5.9	1.0	1.0	-	0.8	1.0
Quercetin 3-glucuronide-glucoside	˂0.1	0.1	-	˂0.1	0.6	-	˂0.1
Epicatechin	2.9	2.0	2.8	0.4	2.3	2.6	1.5
*p*-Coumaroyl quinic acid 1	261.5	175.9	138.5	220.9	14.7	57.8	296.8
*p*-Coumaric acid glycoside	-	8.4	10.5	34.7	-	2.2	9.5
*p*-Coumaroyl quinic acid 2	139.2	112.1	98.0	114.1	14.5	37.1	127.4
Quercetin glucoramnoside	˂0.1	1.5	0.1	-	1.3	˂0.1	0.1
Quercetin pentoside	-	˂0.1	-	-	0.1	-	-
Quercetin pentoside 1	2.1	1.9	2.3	0.8	2.4	1.5	1.5
Ellagic acid	15.1	27.34	28.2	14.2	19.6	26.8	22.0
Quercetin pentoside 2	4.6	5.6	6.2	4.0	3.8	5.8	5.1
Hyperoside	0.6	2.6	1.2	1.3	2.0	1.2	1.4
Quercetin rutinoside (rutin)	24.3	4.1	1.6	2.2	5.8	2.2	3.1
Quercetin 4’-glucuronide	65.7	85.9	34.4	7.8	84.9	49.6	74.0
Isoquercetin	4.3	13.3	6.1	0.4	14.9	3.1	2.8
Quercetin 7-glucuronide	0.1	0.1	0.2	-	˂0.1	˂0.1	-
Quercetin pentoside 3	0.1	39.8	17.3	˂0.1	51.9	27.7	16.2
Quercetin 3-(6”-(3-hydroxy-3-methylglutaryl)hexoside) 1	0.4	3.1	1.0	5.6	5.5	2.8	˂0.1
Kaempferol hexoside	1.6	-	1.1	0.2	-	0.5	0.9
Quercetin hexoside malonate	0.5	-	-	˂0.1	˂0.1	˂0.1	0.5
Isorhamnetin hexoside 1	˂0.1	˂0.1	˂0.1	˂0.1	0.2	˂0.1	˂0.1
Quercetin 3-(6”-(3-hydroxy-3-methylglutaryl)hexoside) 2	-	2.5	0.1	-	2.5	0.5	˂0.1
Isorhamnetin pentoside 1	5.8	-	˂0.1	˂0.1	˂0.1	˂0.1	-
Kaempferol glucuronide	3.6	1.2	0.5	0.2	1.8	0.6	1.4
Isorhamnetin rhamnoside 1	2.4	3.4	3.5	1.1	4.6	2.3	2.7
Dicaffeic acid derivative	7.0	14.2	18.0	-	20.0	7.3	-
Isorhamnetin pentoside 2	˂0.1	1.3	0.2	˂0.1	1.3	˂0.1	0.8
Rhamnetin/isorhamnetin	˂0.1	0.6	0.4	0.2	0.2	0.3	0.2
Ellagic acid acetylarabinoside	37.9	38.9	39.0	13.6	36.8	24.5	28.6
Acetylxyloside of ellagic acid	12.2	6.6	10.1	0.3	2.8	4.5	17.3
Dicaffeoyl quinic acid	1.3	1.2	1.2	1.6	1.2	-	1.4
Isorhamnetin rhamnoside	˂0.1	˂0.1	˂0.1	˂0.1	˂0.1	-	˂0.1
Isorhamnetin rhamnoside 2	1.5	2.6	2.6	0.7	2.6	1.3	2.2
Chlorogenic acid rhamnoside	3.3	2.1	1.6	7.8	1.2	1.1	2.9
Isorhamnetin rhamnoside 3	0.7	1.2	1.2	0.3	1.2	0.4	0.6
Quercetin	0.1	0.8	0.2	0.1	0.2	0.2	0.8
Neochlorogenic acid rhamnoside	1.7	1.3	1.1	3.4	-	-	1.5
Isorhamnetin rhamnoside 6	3.2	3.7	4.1	0.6	4.2	1.6	2.5
Isorhamnetin rhamnoside 7	˂0.1	-	˂0.1	-	˂0.1	-	0.8
Total:	862.8	799.1	913.0	557.1	825.1	584.0	1155.0

**Table 3:** Polyphenols in raspberry stems that grew in home gardens (GR1-GR13), mg%.

Compound	Garden Raspberry												
	GR 1	GR 2	GR 3	GR 4	GR 5	GR 6	GR 7	GR 8	GR 9	GR 10	GR 11	GR 12	GR 13
Dihydroxybenzoic acid hexoside 1	350.7	132.5	-	93.6	124.6	48.2	35.4	55.0	307.7	63.1	-	212.7	49.2
Dihydroxybenzoic acid hexoside 2	58.0	66.3	-	88.7	-	28.8	27.2	28.9	47.4	32.4	-	226.8	32.7
Pentoside of protocatechuic acid	62.2	373.2	134.3	1233.7	1077.7	-	278.3	297.4	101.5	262.1	-	1052.6	402.8
Hydroxybenzoic acid hexoside	31.7	35.4	-	45.3	-	24.2	-	52.0	31.4	-	29.4	111.1	22.4
Procyanidin B(1)	7.2	-	-	4.1	-	4.4	2.8	2.5	3.1	4.8	4.1	3.5	12.6
Dihydroxyferulic acid glycoside	19.7	5.3	-	24.1	9.3	15.4	2.6	27.8	8.4	3.0	-	22.7	-
Catechin	0.8	0.7	0.3	1.7	0.5	2.5	1.4	0.8	2.4	10.8	1.4	7.1	10.1
Chlorogenic acid	6.2	2.0	1.2	11.2	1.2	8.6	2.8	1.4	5.5	1.0	-	1.4	0.8
Procyanidin B(2)	35.1	20.0	2.2	62.6	16.7	72.9	11.7	32.8	27.6	50.6	6.4	53.6	88.5
Procyanidin B(3)	6.9	4.8	-	16.3	5.6	30.3	2.8	7.0	6.0	15.4	8.2	15.3	13.0
Neochlorogenic acid	1.2	1.0	0.9	1.7	-	2.0	1.0	-	1.2	0.8	-	0.9	-
Quercetin 3-glucuronide-glucoside	1.0	˂0.1	˂0.1	7.9	˂0.1	2.7	˂0.1	˂0.1	4.0	0.2	-	1.2	-
Epicatechin	22.3	8.1	1.1	34.7	11.7	46.6	4.8	26.6	15.0	42.2	2.7	54.7	65.6
*p*-Coumaroyl quinic acid 1	15.2	11.6	49.7	18.3	1.2	53.3	21.7	1.5	48.1	6.0	-	7.6	-
*p*-Coumaric acid glycoside	24.2	6.2	4.2	10.6	2.8	27.6	7.8	3.1	52.0	38.3	6.2	51.8	4.6
*p*-Coumaroyl quinic acid 2	15.5	9.2	32.4	12.5	1.7	39.0	17.8	1.8	27.7	5.6	-	3.7	-
Quercetin glucoramnoside	1.9	1.2	0.3	4.2	1.0	1.2	0.1	2.1	2.8	˂0.1	-	0.4	-
Quercetin pentoxoside	0.9	-	-	0.6	0.3	0.9	˂0.1	1.0	1.8	-	-	˂0.1	-
Quercetin pentoside 1	3.4	3.0	0.8	4.5	2.6	1.6	2.1	2.4	3.3	2.4	0.3	6.0	2.2
Ellagic acid	36.9	19.9	35.5	44.4	29.5	24.00	21.4	25.1	26.8	15.1	92.8	67.2	40.2
Quercetin pentoside 2	7.0	4.7	6.10	9.2	6.9	5.6	5.9	6.4	6.6	2.6	14.9	10.0	7.2
Hyperoside	2.7	1.9	2.3	4.0	6.4	2.3	2.1	7.4	3.7	˂0.1	-	˂0.1	0.1
Quercetin rutinoside (rutin)	4.1	1.9	1.5	4.2	22.7	15.1	16.7	8.1	7.5	-	-	-	1.0
Quercetin 4’-glucuronide	61.1	32.8	45.9	105.5	54.6	93.2	54.5	36.8	100.6	-	˂0.1	107.9	1.1
Isoquercetin	5.0	4.8	1.9	28.5	7.1	48.1	5.0	4.3	19.9	8.0	-	10.5	0.2
Quercetin 7-glucuronide	0.6	1.0	-	2.3	1.1	2.5	-	0.8	2.1	57.0	˂0.1	2.8	0.4
Quercetin pentoside 3	21.5	14.6	11.9	33.9	0.6	0.1	5.4	˂0.1	43.6	˂0.1	-	˂0.1	-
Quercetin 3-(6”-(3-hydroxy-3-methyl-glutaryl)hexoside 1	2.2	3.1	1.3	2.4	10.4	2.2	3.5	1.2	4.4	˂0.1	˂0.1	0.4	˂0.1
Kaempferol hexoside	˂0.1	-	-	8.4	1.3	0.8	0.6	0.5	˂0.1	0.5	0.1	3.5	1.8
Quercetin hexoside malonate	1.2	0.1	0.2	5.5	2.8	3.4	2.4	1.8	5.1	1.2	-	1.8	˂0.1
Isorhamnetin hexoside 1	2.3	0.8	-	3.2	2.0	6.7	0.2	1.5	5.3	0.6	˂0.1	3.0	0.6
Quercetin 3-(6”-(3-hydroxy-3-methyl-glutaryl)hexoside 2	0.8	1.8	0.4	3.7	0.3	˂0.1	-	-	˂0.1	-	˂0.1	0.8	-
Isorhamnetin pentoside 1	2.1	1.2	0.6	4.7	2.7	5.1	1.7	2.6	2.4	0.4	0.3	-	-
Kaempferol glucuronide	1.9	0.5	1.0	5.6	3.3	6.7	2.1	2.3	2.6	1.5	-	2.5	˂0.1
Isorhamnetin rhamnoside 1	3.5	˂0.1	1.9	3.4	3.3	1.7	4.1	3.5	2.8	3.1	0.3	6.9	2.5
Dicaffeic acid derivative	29.7	59.0	10.2	19.9	5.7	37.9	4.0	32.2	30.8	10.4	4.0	66.4	6.4
Isorhamnetin pentoside 2	0.4	˂0.1	0.8	˂0.1	-	-	-	˂0.1	0.5	-	-	˂0.1	0.8
Rhamnetin/isorhamnetin	0.6	0.3	1.4	1.4	0.9	1.4	0.5	0.4	0.5	0.2	3.9	1.9	1.4
Ellagic acid acetylarabinoside	31.1	21.8	18.7	50.5	28.6	28.6	46.0	53.1	34.1	12.9	3.1	67.6	19.4
Acetylxyloside of ellagic acid	42.3	19.7	6.7	43.5	30.3	30.1	44.8	35.0	48.6	4.2	1.3	34.8	25.3
Dicaffeoyl quinic acid	2.3	1.5	1.2	4.2	1.5	1.6	1.4	1.2	2.8	-	-	1.2	-
Isorhamnetin rhamnoside	0.7	0.3	-	6.6	1.6	7.6	˂0.1	0.6	2.8	0.1	˂0.1	2.1	1.3
Isorhamnetin rhamnoside 2	3.1	1.2	1.5	3.6	2.3	0.9	2.6	2.5	2.67	2.1	0.3	8.5	1.9
Chlorogenic acid rhamnoside	2.2	1.3	1.6	3.7	1.1	3.9	2.2	-	2.8	-	-	1.1	-
Isorhamnetin rhamnoside 3	1.5	0.5	0.3	1.0	0.6	0.3	1.3	1.2	1.0	0.6	0.1	4.0	1.2
Quercetin	1.7	0.1	0.6	1.8	0.7	0.9	0.2	0.1	1.9	0.2	0.1	0.4	˂0.1
Neochlorogenic acid rhamnoside	1.5	1.2	-	1.6	-	1.8	1.4	-	1.6	-	-	-	-
Isorhamnetin rhamnoside 6	3.3	2.5	1.78	3.2	2.0	1.3	3.3	2.9	3.4	3.5	0.4	7.5	-
Isorhamnetin rhamnoside 7	˂0.1	˂0.1	0.2	3.7	˂0.1	-	˂0.1	˂0.1	˂0.1	˂0.1	˂0.1	0.5	2.4
Total:	936.9	878.6	382.6	2089.6	1486.9	743.8	653.8	775.4	1063.4	662.8	180.5	2246.2	819.2

**Table 4:** Polyphenols in the raspberry stems that grew in the wild (WR1-WR13), mg%.

Compound	Wild Raspberry												
	WR 1	WR 2	WR 3	WR 4	WR 5	WR 6	WR 7	WR 8	WR 9	WR 10	WR 11	WR 12	WR 13
Dihydroxybenzoic acid hexoside 1	141.0	23.4	-	98.5	123.3	106.1	-	74.0	15.9	22.7	55.7	17.2	-
Dihydroxybenzoic acid hexoside 2	36.5	76.4	176.5	59.8	36.4	74.8	-	84.6	-	19.5	21.3	13.8	32.0
Pentozide of protocatechuic acid	456.9	130.6	530.1	517.7	742.6	793.5	199.4	294.1	457.3	185.3	597.1	224.2	175.9
Hydroxybenzoic acid hexoside	-	72.3	300.8	41.0	-	40.8	-	-	-	-	-	-	-
Procyanidin B(1)	2.1	-	2.3	-	-	-	3.6	-	5.7	3.0	1.7	-	-
Dihydroxyferulic acid glycoside	9.8	1.4	10.2	19.1	-	-	-	1.9	-	1.5	-	2.0	-
Catechin	0.5	0.8	0.5	1.4	0.7	0.3	1.6	4.1	1.5	6.0	0.7	0.8	0.6
Chlorogenic acid	6.1	1.4	2.4	3.2	1.0	0.8	-	1.2	-	1.4	0.8	2.4	2.1
Procyanidin B(2)	18.2	4.5	25.4	27.9	6.7	9.5	56.8	14.6	93.0	24.3	20.5	4.2	2.8
Procyanidin B(3)	8.2	3.2	7.8	9.9	3.6	4.2	9.6	7.1	15.6	9.8	4.7	3.0	2.2
Neochlorogenic acid	1.8	-	0.9	0.9	-	-	-	-	-	-	-	0.9	-
Quercetin 3-glucuronide-glucoside	˂0.1	˂0.1	1.2	3.6	˂0.1	˂0.1	-	0.1	-	˂0.1	-	-	-
Epicatechin	19.1	3.0	13.6	25.8	4.2	3.0	18.1	17.8	81.9	17.8	7.9	6.5	2.1
*p*-Coumaroyl quinic acid 1	65.8	15.4	2.7	2.3	4.3	-	-	7.4	-	16.4	-	37.4	109.8
*p*-Coumaric acid glycoside	-	6.3	11.3	39.4	48.4	2.1	43.2	6.1	21.9	36.5	16.5	39.5	78.3
*p*-Coumaroyl quinic acid 2	62.9	9.4	2.5	3.1	3.7	-	-	3.7	-	12.3	-	29.2	60.0
Quercetin glucoramnoside	-	˂0.1	0.5	8.4	-	˂0.1	-	-	0.1	˂0.1	˂0.1	-	-
Quercetin pentoxoside	0.4	˂0.1	˂0.1	2.7	-	-	-	˂0.1	˂0.1	˂0.1	-	-	-
Quercetin pentoside 1	1.2	2.1	3.1	3.5	1.1	2.6	4.3	1.3	2.2	2.6	2.5	1.5	1.0
Ellagic acid	24.9	18.6	31.2	21.0	14.6	27.2	82.8	26.8	48.4	24.2	31.3	27.2	15.9
Quercetin pentoside 2	1.4	1.9	6.5	4.8	4.0	4.9	23.1	4.7	7.2	5.0	6.4	5.4	2.5
Hyperoside	5.3	6.3	16.6	12.2	3.5	7.4	0.1	2.8	0.5	˂0.1	-	5.9	6.2
Quercetin rutinoside (rutin)	4.1	4.9	10.2	6.1	2.5	4.2	-	1.7	-	2.1	0.5	4.5	4.0
Quercetin 4’-glucuronide	17.5	40.0	63.7	83.1	12.5	21.3	0.9	67.2	3.8	67.4	12.7	6.7	16.9
Isoquercetin	4.0	4.9	7.1	16.5	2.3	2.8	1.9	4.7	3.0	14.9	1.6	2.3	1.9
Quercetin 7-glucuronide	0.8	-	1.5	2.3	˂0.1	0.4	1.4	-	1.4	-	1.0	˂0.1	-
Quercetin pentoside 3	˂0.1	˂0.1	˂0.1	22.8	3.9	˂0.1	-	16.6	˂0.1	-	-	0.1	˂0.1
Quercetin 3-(6”-(3-hydroxy-3-methyl-glutaryl)hexoside) 1	5.3	3.9	24.6	9.1	0.4	6.9	0.2	2.1	0.1	˂0.1	˂0.1	2.4	8.9
Kaempferol hexoside	˂0.1	1.0	0.5	0.4	0.1	0.1	-	˂0.1	0.9	1.3	3.0	1.6	1.2
Quercetin hexoside malonate	˂0.1	1.3	1.5	3.5	0.5	0.8	˂0.1	0.6	˂0.1	2.8	˂0.1	0.3	0.1
Isorhamnetin hexoside 1	3.0	˂0.1	˂0.1	1.6	0.2	0.7	3.7	0.1	3.0	˂0.1	0.7	˂0.1	˂0.1
Quercetin 3-(6”-(3-hydroxy-3-methyl-glutaryl)hexoside) 2	˂0.1	-	0.8	4.3	˂0.1	˂0.1	-	0.4	˂0.1	2.6	0.8	-	˂0.1
Isorhamnetin pentoside 1	0.8	1.4	1.5	4.8	1.5	0.9	1.9	1.1	˂0.1	-	-	1.1	0.7
Kaempferol glucuronide	0.8	2.2	2.5	7.3	1.0	0.8	˂0.1	1.5	0.4	1.5	0.3	0.6	1.1
Isorhamnetin rhamnoside 1	1.9	4.0	2.9	3.5	2.8	3.7	4.6	2.2	1.0	4.0	3.0	2.3	1.8
Dicaffeic acid derivative	38.5	7.4	9.6	41.1	11.1	12.5	12.3	-	2.2	4.9	23.5	10.0	2.7
Isorhamnetin pentoside 2	0.1	˂0.1	0.4	1.9	1.0	0.1	1.0	0.3	0.5	˂0.1	2.8	0.8	˂0.1
Rhamnetin/isorhamnetin	0.8	0.2	0.4	0.9	0.3	1.1	2.7	0.2	2.9	0.6	1.6	0.6	1.0
Acetylarabinoside of ellagic acid	26.8	48.7	48.1	40.3	39.0	43.2	80.8	34.4	26.2	38.9	41.0	24.4	13.2
Acetylxyloside of ellagoic acid	16.6	14.0	34.3	19.9	11.7	19.2	69.1	12.8	25.7	9.9	23.2	4.8	˂0.1
Dicaffeoyl quinic acid	2.2	-	2.3	1.7	-	-	-	1.1	-	-	-	1.1	-
Isorhamnetin rhamnoside	1.1	˂0.1	2.1	3.6	˂0.1	0.8	1.8	0.1	1.2	˂0.1	0.3	-	-
Isorhamnetin rhamnoside 2	1.5	2.8	2.2	3.8	2.7	2.8	2.9	1.8	0.5	3.4	3.2	1.9	1.3
Chlorogenic acid rhamnoside	5.4	1.3	1.4	1.1	-	-	-	-	-	-	-	1.4	1.3
Isorhamnetin rhamnoside 3	0.5	1.2	0.9	1.0	0.9	1.6	2.3	0.6	0.4	1.3	0.8	0.5	0.4
Quercetin	0.8	0.2	0.6	0.9	-	˂0.1	0.2	0.3	0.3	0.4	˂0.1	0.2	0.1
Neochlorogenic acid rhamnoside	2.8	-	-	-	-	-	-	-	-	-	-	-	-
Isorhamnetin rhamnoside 6	2.0	4.6	3.4	3.1	3.0	4.2	3.9	3.0	0.6	5.1	2.1	1.6	1.7
Isorhamnetin rhamnoside 7	-	-	˂0.1	2.0	1.9	˂0.1	˂0.1	0.8	-	˂0.1	1.4	0.4	˂0.1
Total:	999.3	520.8	1368.4	1192.8	1097.4	1205.2	634.1	705.9	825.0	549.1	890.3	490.5	549.7

**Table 5:** Origin of the samples.

Sample	Origin
CR 1 (‘Glen Ample’)	EMÜ Centre for Horticultural Research, Polli, Karksi parish, Viljandi County
CR 2 (‘Tomo’)	EMÜ Centre for Horticultural Research, Polli, Karksi parish, Viljandi County
CR 3 (‘Siveli’)	EMÜ Centre for Horticultural Research, Polli, Karksi parish, Viljandi County
CR 4 (‘Espe’)	EMÜ Centre for Horticultural Research, Polli, Karksi parish, Viljandi County
CR 5 (‘Aita’)	EMÜ Centre for Horticultural Research, Polli, Karksi parish, Viljandi County
CR 6 (‘Helkal’)	EMÜ Centre for Horticultural Research, Polli, Karksi parish, Viljandi County
CR 7 (‘Alvi’)	EMÜ Centre for Horticultural Research, Polli, Karksi parish, Viljandi County
GR 1 (‘Tomo’)	Simmi farm, Kivilõppe village, Tarvastu parish, Viljandi County
GR 2	Iisaku, Iisaku parish, Ida-Viru County
GR 3	Kadarbiku village, Taebla parish, Lääne County
GR 4	Kadarbiku village, Taebla parish, Lääne County
GR 5	Vanamõisa farm, Kolila village, Ridala parish, Lääne County
GR 6 (‘Herbert’)	Soe village, Tarvastu parish, Viljandi County
GR 7	Soe village, Tarvastu parish, Viljandi County
GR 8	Paeküla, Märjamaa parish, Rapla County
GR 9 (‘Tomo’)	Rüssa farm, Kivilõppe village, Tarvastu parish, Viljandi County
GR 10	Raudtee street, Tõrva city, Valga County
GR 11 (‘Ottawa’)	Raudtee street, Tõrva city, Valga County
GR 12 (‘Polka’)	Rebase Street, Tartu, Tartu County
GR 13	Rebase Street, Tartu, Tartu County
WR 1	Paju otsas, Simmi farm, Kivilõppe village, Tarvastu parish, Viljandi County
WR 2	Simmi Forest, Kivilõppe village, Tarvastu parish, Viljandi County
WR 3	Härma quarry, Helme parish, Valga county
WR 4	Palu mets, Järveküla, Tarvastu parish, Viljandi County
WR 5	Iisaku Forest, Iisaku Parish, Ida-Viru County
WR 6	Vanamõisa lakeside, Tõrva city, Valga county
WR 7	Kadarbiku village, Taebla parish, Lääne County
WR 8	Vasara village, Viljandi parish, Viljandi County
WR 9	Kolila village, Ridala parish, Lääne County
WR 10	Lake Võrtsjärve, Kivilõppe village, Tarvastu parish, Viljandi County
WR 11	Rüssa Forest, Kivilõppe village, Tarvastu parish, Viljandi County
WR 12	Rulli village, Põdrala parish, Valga County
WR 13	Ahimäe village, Karksi parish, Viljandi County

CR—cultivar raspberry; GR—garden raspberry; WR—wild raspberry.

**Figure A1:**
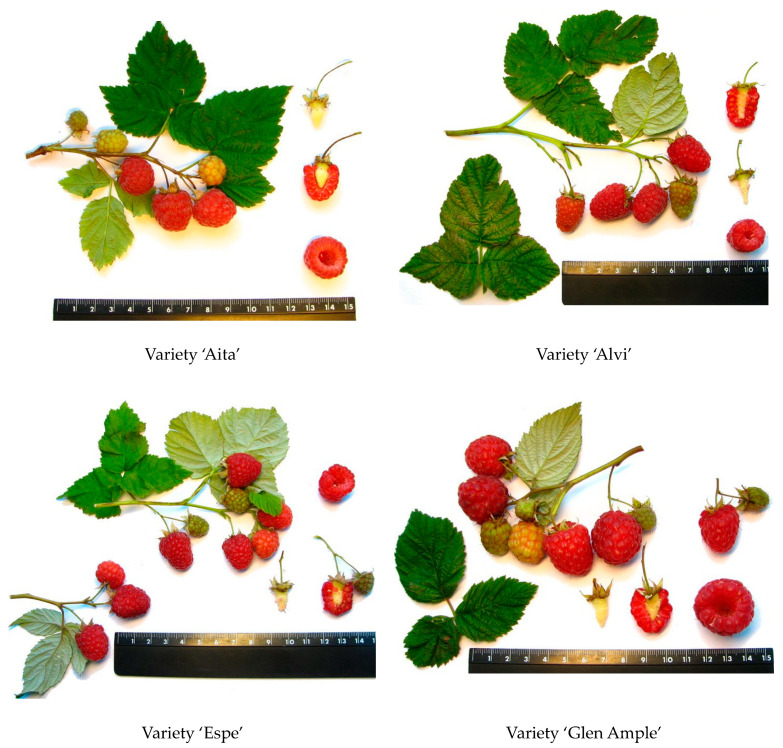

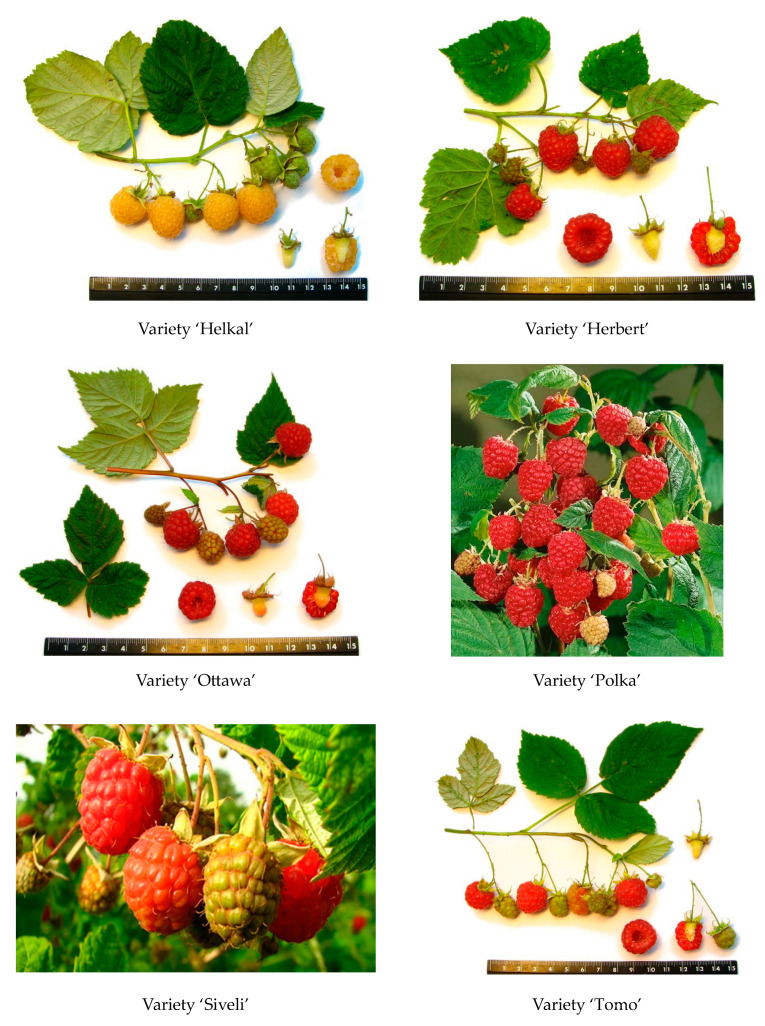
Images of the studied raspberry varieties.

## Data Availability

The data supporting the results of this study can be obtained from the corresponding authors upon reasonable request.

## Appendix A

**Table A1:** Polyphenols in different parts of the raspberry stems, mg%.

Compound	GR 12 I	GR 12 II	GR 12 III	GR 12 IV	GR 12 V	GR 13 I	GR 13 II	GR 13 III	GR 13 IV	OCT I	OCT II	OCT III	OCT IV	OCT V
Dihydroxybenzoic acid hexoside 1	47.9	56.1	212.7	139.2	38.5	49.2	146.8	55.8	34.5	-	-	-	-	-
Dihydroxybenzoic acid hexoside 2	18.1	-	22.6	67.1	-	32.6	28.6	21.1	16.4	-	-	-	-	-
Pentozide of protocatechuic acid	98.4	85.5	1052.6	516.4	173.9	402.8	292.2	170.6	72.4	-	-	-	-	-
Hydroxybenzoic acid hexoside	8.2	-	11.1	42.5	-	22.4	17.8	20.6	8.7	-	-	-	-	-
Procyanidin B(1)	3.5	3.2	3.5	8.2	6.2	12.6	9.8	5.9	4.2	5.0	5.1	2.5	-	-
Dihydroxyferulic acid glycoside	-	-	22.7	14.3	1.5	-	-	-	-	-	4.0	2.1	-	-
Catechin	1.9	1.7	7.1	16.3	6.3	10.1	5.4	4.8	4.1	23.9	8.3	2.4	2.5	2.6
Chlorogenic acid	-	-	1.4	1.0	-	0.8	-	-	-	2.4	1.4	-	-	-
Procyanidin B(2)	69.8	64.4	53.6	150.2	110.7	88.5	85.5	85.2	83.5	109.4	91.3	53.2	59.2	80.0
Procyanidin B(3)	4.7	3.5	15.3	32.1	7.8	13.0	9.3	8.9	6.9	13.1	9.2	5.2	5.3	7.6
Neochlorogenic acid	-	-	0.9	-	-	-	-	-	-	0.9	-	-	-	-
Quercetin 3-glucuronide-glucoside	-	-	1.2	0.5	-	-	-	-	-	0.1	˂0.1	-	-	-
Epicatechin	60.1	52.1	54.7	124.5	87.5	65.6	64.0	64.7	58.7	42.4	38.0	25.0	25.0	31.0
*p*-Coumaroyl quinic acid 1	-	-	7.6	2.0	-	-	-	-	-	46.9	18.1	4.0	-	-
*p*-Coumaric acid glycoside	2.4	2.4	51.8	19.7	4.6	4.6	2.5	2.0	2.0	23.4	14.3	4.6	2.6	1.9
*p*-Coumaroyl quinic acid 2	-	-	3.7	1.3	-	-	-	-	-	31.4	13.0	2.3	1.3	-
Quercetin glucorhamnoside	-	-	0.4	˂0.1	-	-	-	-	-	0.2	˂0.1	-	-	-
Quercetin pentoside	-	-	˂0.1	˂0.1	-	-	-	-	-	˂0.1	0.2	-	-	-
Quercetin pentoside 1	3.1	2.5	6.0	4.5	3.4	2.2	2.2	2.2	2.0	0.7	0.9	1.3	1.1	1.1
Ellagic acid	19.9	13.1	67.2	47.5	27.6	40.2	21.8	24.0	18.2	10.8	12.1	14.9	15.0	11.9
Quercetin pentoside 2	3.7	3.0	10.0	6.6	4.7	7.2	5.4	5.5	4.8	2.0	2.0	2.4	2.2	2.3
Quercetin rutinoside dicaffeic acid	-	-	-	-	-	-	-	-	-	0.6	0.5	-	-	1.0
Hyperoside	0.6	0.2	10.5	17.7	2.0	1.0	0.5	˂0.1	˂0.1	26.9	1.8	˂0.1	-	-
Quercetin 4’-glucuronide	0.1	˂0.1	108.0	82.3	6.0	1.1	0.1	˂0.1	˂0.1	43.1	24.2	1.8	˂0.1	˂0.1
Isoquercetin	˂0.1	˂0.1	˂0.1	˂0.1	˂0.1	0.2	˂0.1	˂0.1	˂0.1	47.0	3.2	0.4	˂0.1	˂0.1
Quercetin 7-glucuronide	0.1	˂0.1	2.8	2.4	0.4	0.4	˂0.1	˂0.1	˂0.1	0.5	0.1	˂0.1	˂0.1	˂0.1
Quercetin pentoside 3		-	˂0.1	0.1	˂0.1	-	-	-	-	12.3	6.9	1.1	˂0.1	-
Quercetin 3-(6”-(3-hydroxy-3-methyl-glutaryl)hexoside) 1	˂0.1	˂0.1	0.8	0.3	˂0.1	˂0.1	-	˂0.1	-	0.6	0.3	-	-	-
Kaempferol hexoside	1.3	1.1	3.5	1.1	1.6	1.8	1.6	2.0	1.6	˂0.1	-	-	-	-
Quercetin hexoside malonate	-	-	0.1	0.1	-	˂0.1	-	-	-	14.8	0.7	˂0.1	-	-
Isorhamnetin hexoside 1	˂0.1	˂0.1	3.0	2.2	0.2	0.6	˂0.1	˂0.1	˂0.1	1.1	0.6	˂0.1	˂0.1	˂0.1
Quercetin 3-(6”-(3-hydroxy-3-methyl-glutaryl)hexoside) 2	˂0.1	-	0.4	0.3	˂0.1	-	-	-	-	0.2	˂0.1	-	-	-
Isorhamnetin pentoside 1	-	-	-	1.2	-	-	-	-	-	0.3	0.3	0.4	0.2	0.3
Kaempferol glucuronide	˂0.1	-	2.5	1.8	˂0.1	˂0.1	-	-	-	7.5	0.1	-	-	-
Isorhamnetin rhamnoside 1	0.6	0.6	2.7	2.1	0.8	2.5	2.0	2.2	2.2	13.1	0.9	0.8	0.6	0.4
Dicaffeic acid derivative	7.1	3.0	66.4	28.4	16.0	6.4	6.6	3.9	-	-	-	-	-	-
Isorhamnetin pentoside 2	0.1	˂0.1	˂0.1	˂0.1	0.1	0.8	0.5	0.7	0.7	˂0.1	0.1	0.1	0.2	0.2
Rhamnetin/isorhamnetin	0.3	0.1	1.9	0.8	0.5	1.4	0.7	0.8	0.6	0.2	0.3	0.3	0.2	0.2
Acetylarabinoside of ellagoic acid	25.7	22.3	115.8	71.3	37.3	19.4	16.8	18.3	12.7	21.6	15.1	16.8	13.4	11.0
Acetylxyloside of ellagic acid	15.8	13.8	67.6	42.8	24.9	25.3	29.8	31.6	27.3	17.5	14.9	12.1	6.1	8.1
Dicaffeoyl quinic acid	-	-	1.2	-	-	-	-	-	-	1.4	1.2	-	-	-
Isorhamnetin rhamnoside	-	-	2.1	1.5	-	1.3	0.5	-	-	0.2	-	-	-	-
Isorhamnetin rhamnoside 2	1.5	1.2	8.5	4.6	2.7	1.9	1.5	1.9	1.2	1.1	0.7	0.4	0.3	0.2
Chlorogenic acid rhamnoside	-	-	1.1	-	-	-	-	-	-	2.5	1.7	-	-	-
Isorhamnetin rhamnoside 3	0.8	0.5	4.0	2.6	1.7	1.2	1.0	1.3	0.9	˂0.1	˂0.1	˂0.1	˂0.1	˂0.1
Quercetin	-	-	0.4	5.3	0.5	0.2	0.1	-	-	0.1	0.3	˂0.1	-	-
Neochlorogenic acid rhamnoside	-	-	-	-	-	-	-	-	-	1.4	1.8	1.1	-	-
Isorhamnetin rhamnoside 6	0.1	˂0.1	1.9	0.9	0.3	˂0.1	˂0.1	˂0.1	˂0.1	2.2	˂0.1	˂0.1	˂0.1	-
Isorhamnetin rhamnoside 7	2.5	2.1	9.9	7.3	3.6	2.4	1.8	2.4	1.7	-	1.4	1.0	0.6	0.7
Total:	2089.6	1486.9	936.9	878.6	382.6	819.3	754.6	536.2	365.3	529.1	295.0	156.8	135.9	160.4

## Appendix B

**Table A2:** Positive correlations among the contents of individual compounds in the raspberry stems.

Pairs of Compounds	Correlation Coefficient, r
Procyanidin-catechin	
Procyanidin B(1)-Epicatechin	0.60
Procyanidin B(1)-Procyanidin B(2)	0.64
Procyanidin B(3)-Epicatechin	0.76
Procyanidin B(2)-Procyanidin B(3)	0.78
Procyanidin B(2)-Epicatechin	0.93
Procyanidin-flavonols	
Procyanidin B(2)-Isorhamnetin rhamnoside 7	0.60
Procyanidin B(3)-Isorhamnetin hexoside 1	0.62
Procyanidin B(3)-Isorhamnetin rhamnoside	0.71
Procyanidin B(3)-Isoquercetin	0.73
Benzoic acid derivatives-Ellagic acid derivatives	
Dihydroxybenzoic acid hexoside 2-Ellagic acid	0.62
Ellagic acid acetylarabinoside-Acetylxyloside of ellagic acid	0.64
Dihydroxybenzoic acid hexoside 2-Hydroxybenzoic acid hexoside	0.70
Hydroxycinnamic acids derivatives	
Chlorogenic acid-Dicaffeoyl quinic acid	0.70
Chlorogenic acid-Neochlorogenic acid	0.79
Chlorogenic acid-Chlorogenic acid rhamnoside	0.73
Chlorogenic acid rhamnoside-Neochlorogenic acid rhamnoside	0.97
*p*-Coumaroyl quinic acid 1-*p*-coumaroyl quinic acid 2	0.97
Hydroxycinnamic acids derivatives-catechin–procyanidin	
Dihydroferulic acid glycoside-Epicatechin	0.61
Dihydroferulic acid glycoside-Procyanidin B(2)	0.63
Dihydroxybenzoic acid derivatives-flavonols	
Dihydroxybenzoic acid hexoside 2-Isorhamnetin rhamnoside 6	0.62
Dihydroxybenzoic acid hexoside 2-Isorhamnetin rhamnoside 1	0.63
Dihydroxybenzoic acid hexoside 1-Quercetin	0.64
Dihydroxybenzoic acid hexoside 2-Isorhamnetin rhamnoside 2	0.64
Dihydroxybenzoic acid hexoside 2-Quercetin pentoside 1	0.66
Dihydroxybenzoic acid hexoside 2-Isorhamnetin rhamnoside 3	0.66
Dihydroxybenzoic acid hexoside 2-Quercetin pentoside 3	0.72
Hydroxybenzoic acid hexoside-Hyperoside	0.78
Hydroxybenzoic acid hexoside-Quercetin 3-(6″-(3-hydroxy-3-methylglutaryl)hexoside) 1	0.84
Ellagic acid derivatives-flavonols	
Quercetin 3-glucuronide-glucoside-Acetylxyloside of ellagic acid	0.61
Quercetin hexoside malonate-Acetylxyloside of ellagic acid	0.64
Acetylxyloside of ellagic acid-Isorhamnetin rhamnoside 7	0.65
Quercetin pentoside 1-Acetylxyloside of ellagic acid	0.66
Ellagic acid acetylarabinoside-Isorhamnetin rhamnoside 6	0.70
Quercetin pentoside 1-Ellagic acid acetylarabinoside	0.71
Ellagic acid acetylarabinoside-Isorhamnetin rhamnoside 3	0.76
Isorhamnetin rhamnoside 1-Ellagic acid acetylarabinoside	0.78
Ellagic acid-Rhamnetin/isorhamnetin	0.87
Ellagic acid-Quercetin pentoside 2	0.88
Hydroxycinnamic acids derivatives-flavonols	
Dihydroferulic acid glycoside-Isorhamnetin rhamnoside 7	0.60
Neochlorogenic acid-Quercetin hexoside malonate	0.60
Quercetin 3-(6″-(3-hydroxy-3-methylglutaryl)hexoside) 2-Neochlorogenic acid rhamnoside	0.60
Dihydroferulic acid glycoside-Rhamnetin/isorhamnetin	0.61
Chlorogenic acid-Isorhamnetin rhamnoside 7	0.61
Chlorogenic acid-Quercetin	0.62
Neochlorogenic acid-Isorhamnetin rhamnoside	0.64
Chlorogenic acid rhamnoside-Isorhamnetin rhamnoside 7	0.64
Chlorogenic acid-Isorhamnetin pentoside 1	0.64
*p*-Coumaroyl quinic acid 1-Isorhamnetin hexoside 1	0.68
Dihydroferulic acid glycoside-Isorhamnetin pentoside 1	0.68
Ellagic acid acetylarabinoside-Isorhamnetin rhamnoside 2	0.68
Neochlorogenic acid-Isorhamnetin hexoside 1	0.70
Quercetin 3-(6″-(3-hydroxy-3-methylglutaryl)hexoside) 1-Neochlorogenic acid rhamnoside	0.68
Chlorogenic acid-Isorhamnetin hexoside 1	0.73
Quercetin hexoside malonate-Dicaffeoyl quinic acid	0.73
*p*-Coumaric acid glycoside-Quercetin pentoxoside	0.76
Dicafeoyl quinic acid-Quercetin	0.78
Kempferol glycoside-Dicafeoyl quinic acid	0.78
Chlorogenic acid-Isorhamnetin rhamnoside	0.78
Chlorogenic acid-Quercetin 3-glucuronide-glucoside	0.81
Neochlorogenic acid-Isorhamnetin rhamnoside 7	0.87
Quercetin 3-glucuronide-glucoside-Dicafeoyl quinic acid	0.86
Dicafeoyl quinic acid-Isorhamnetin rhamnoside 7	0.94
Neochlorogenic acid rhamnoside-Isorhamnetin rhamnoside 7	1.00
Flavonols-flavonols	
Quercetin pentoside 1-Kempferol glycoside	0.60
Quercetin 4’-glucuronide-Isorhamnetin rhamnoside	0.62
Quercetin glucoramnoside-Isorhamnetin rhamnoside 7	0.64
Quercetin pentoxoside-Hyperoside	0.64
Isoquercetin-Isorhamnetin pentoside 1	0.64
Isoquercetin-Isorhamnetin rhamnoside 7	0.64
Quercetin glucorhamnoside-Isorhamnetin pentoside 1	0.65
Quercetin 4’-glucuronide-Kempferol glucuronide	0.65
Isorhamnetin rhamnoside-Isorhamnetin rhamnoside 7	0.65
Quercetin 3-glucuronide-glucoside-Quercetin 3-(6″-(3-hydroxy-3-methylglutaryl)hexoside) 2	0.66
Quercetin 4’-glucuronide-Isorhamnetin pentoside 1	0.66
Quercetin 3-(6″-(3-hydroxy-3-methylglutaryl)hexoside) 2-Kempferol glucuronide	0.67
Quercetin 3-glucuronide-glucoside-Kempferol glucuronide	0.68
Quercetin 4’-glucuronide-Isoquercetin	0.68
Isoquercetin-Isorhamnetin hexoside 1	0.68
Quercetin hexoside malonate-Isorhamnetin hexoside 1	0.68
Quercetin pentoside 1-Isorhamnetin rhamnoside 6	0.69
Quercetin hexoside malonate-Kempferol glucuronide	0.69
Quercetin hexoside malonate-Quercetin	0.69
Quercetin 3-glucuronide-glucoside-Quercetin	0.70
Quercetin glucorhamnoside-Isorhamnetin pentoside 2	0.70
Quercetin pentoside 3-Isorhamnetin rhamnoside 7	0.70
Kempferol glycoside-Quercetin	0.70
Quercetin pentoside 2-Rhamnetin/isorhamnetin	0.71
Quercetin 4’-glucuronide-Quercetin hexoside malonate	0.71
Quercetin hexoside malonate-Quercetin 3-(6″-(3-hydroxy-3-methylglutaryl)hexoside) 2	0.71
Quercetin glucoramnoside-Kempferol glucuronide	0.72
Isorhamnetin hexoside 1-Isorhamnetin rhamnoside	0.72
Quercetin 3-glucuronide-glucoside-Isorhamnetin pentoside 1	0.73
Isoquercetin-Quercetin hexoside malonate	0.73
Quercetin 3-glucuronide-glucoside-Isorhamnetin rhamnoside	0.74
Isoquercetin-Kempferol glucuronide	0.75
Quercetin 3-(6″-(3-hydroxy-3-methylglutaryl)hexoside) 2-Isorhamnetin rhamnoside	0.75
Isorhamnetin pentoside 1-Isorhamnetin pentoside 2	0.75
Quercetin hexoside malonate-Isorhamnetin rhamnoside	0.76
Isorhamnetin rhamnoside-Isorhamnetin rhamnoside 7	0.77
Quercetin pentoside 1-Isorhamnetin rhamnoside 1	0.78
Quercetin pentoside 1-Isorhamnetin rhamnoside 2	0.78
Quercetin pentoside 1-Isorhamnetin rhamnoside 3	0.78
Quercetin 3-glucuronide-glucoside-Kempferol glycoside	0.79
Quercetin glucoramnoside-Quercetin pentoxoside	0.80
Quercetin 3-(6″-(3-hydroxy-3-methylglutaryl)hexoside) 2-Isorhamnetin rhamnoside 7	0.81
Hyperoside-Quercetin 3-(6″-(3-hydroxy-3-methylglutaryl)hexoside) 1	0.82
Isoquercetin-Quercetin 3-(6″-(3-hydroxy-3-methylglutaryl)hexoside) 2	0.83
Isorhamnetin pentoside 1-Isorhamnetin rhamnoside 7	0.83
Quercetin glucoramnoside-Quercetin 3-(6″-(3-hydroxy-3-methylglutaryl)hexoside) 2	0.84
Kempferol glucuronide-Isorhamnetin rhamnoside	0.84
Isorhamnetin rhamnoside 2-Isorhamnetin rhamnoside 6	0.84
Quercetin hexoside malonate-Isorhamnetin rhamnoside 7	0.85
Quercetin 3-(6″-(3-hydroxy-3-methylglutaryl)hexoside) 2-Isorhamnetin pentoside 1	0.85
Isorhamnetin rhamnoside 7-Isorhamnetin rhamnoside 6	0.85
Isorhamnetin pentoside 1-Isorhamnetin rhamnoside	0.86
Isorhamnetin pentoside 1-Kempferol glucuronide	0.87
Isorhamnetin rhamnoside 1-Isorhamnetin rhamnoside 2	0.88
Isorhamnetin rhamnoside 1-Isorhamnetin rhamnoside 3	0.88
Isorhamnetin rhamnoside 2-Isorhamnetin rhamnoside 3	0.89
Isorhamnetin rhamnoside 1-Isorhamnetin rhamnoside 6	0.91
Quercetin 3-glucuronide-glucoside-Quercetin hexoside malonate	0.92
Isoquercetin-Isorhamnetin rhamnoside	0.92
Quercetin-Isorhamnetin rhamnoside 7	0.92
Quercetin 3-glucuronide-glucoside-Isorhamnetin rhamnoside 7	0.97

**Table A3:** Negative correlations among the contents of individual compounds in the Raspberry stems.

Pairs of Compounds	Correlation Coefficient, r
Hydroxybenzoic acid hexoside-Isorhamnetin rhamnoside 7	−0.74
Neochlorogenic acid rhamnoside-Isorhamnetin rhamnoside 6	−0.78
Neochlorogenic acid-Quercetin pentoside	−0.80
Isorhamnetin rhamnoside 1-Neochlorogenic acid rhamnoside	−0.82
Quercetin pentoxoside-Isorhamnetin rhamnoside 7	−1.0

## Appendix C

**Table A4:** Short descriptions of cultivated varieties of *Rubus idaeus*.

Variety	Place of Selection	Cross Made	Fruit	Bush
Aita	Polli Horticultural Research Centre, Estonia	Seedlings of Johannes Parksepp Nr. 2–64–24 × ‘Glen Clova’.	Early maturing, light red, big (average 3.7 g), round, druplets cohering firmly, easy cropping	Moderately growing, young canes, light green with weak spines; fruiting canes are light brown.
Alvi		Seedling of 67-60-12 × ‘Novost Kuzmina’.	ather late, dark red, bright, big (average 3.5 g), conical, druplets cohering firmly, with good quality	Moderately growing, young canes light green with few spines; fruiting canes are greyish brown.
Helkal		Seedlings of the breeder 67-60-12 (‘Golden Queen’ × ‘Spirina Belaja’) × ‘Novost Kuzmina’	Midseason, orange yellow, big (average 3.5 g), round conical, druplets cohering firmly	Moderately strong, producing numerous erect canes, which are light green, covered thickly with spines; fruiting canes are light brown.
Espe		‘Deutschland’ and ‘Novost Kuzmina’	Red, blunt-cone-shaped fruits are medium ripe and medium in size (average of 2.5 g). The partial fruits are well joined and firmly attached to the base of the flower.	Erect stems are high and their stems slightly curled. Light green shoots are strong, have single weak spikes. The second-year stems are light brown.
Toмo		‘Superlative’ × ‘Novost Kuzmina’	Midseason, dark red, medium, round or oblate, and druplets that cohere firmly.	Moderately growing, producing medium or numerous erect canes, which are light green with few weak spines; the fruiting cane is light brown with a grey tinge.
Siveli		‘Golden Queen’ × ‘Spirina belaja’ × ‘Novost kuzmina’	Red fruits are medium-sized and round or broad–round; partial fruits are well joined, relatively resistant to collapse;	The height of the erect stem is average. The shoots are light green with weak spikes, which are more sparsely located at the top of the stem. In the second year, the stems are light brown with a grayish tinge,
Polka			dark red are large and conical.	medium-growing, upright, and high-yielding.
Glen Ample	Scotland	Crossbreeding ‘Glen Rosa’ and ‘Meeker’	Large, conical, bright red berries that can weigh up to 3gm.	Stems are strong, erect, and spine-free.
Herbert	Canada		The fruits are round.	The growth of stems is moderate, shoots have a slightly purple bark, and on the branch many sharp spikes are only located in the top part. Bright red spikes are very sharp.
